# Effects of SiO _2_ Incorporation on the Flexural Properties of a Denture Base Resin: An In Vitro Study

**DOI:** 10.1055/s-0041-1732806

**Published:** 2021-08-24

**Authors:** Sara T. Alzayyat, Ghadah A. Almutiri, Jawhara K. Aljandan, Raneem M. Algarzai, Soban Q. Khan, Sultan Akhtar, Ijlal Shahrukh Ateeq, Mohammed M. Gad

**Affiliations:** 1 Department of Dental Education, College of Dentistry, Imam Abdulrahman Bin Faisal University, Dammam, Saudi Arabia; 2 Department of Dental Education, College of Dentistry, Imam Abdulrahman Bin Faisal University, Dammam, Saudi Arabia; 3 Department of Biophysics, Institute for Research and Medical Consultations, Imam Abdulrahman Bin Faisal University, Dammam, Saudi Arabia; 4 Biomedical Engineering department, College of Engineering, Imam Abdulrahman Bin Faisal University, Dammam, Saudi Arabia; 5 Department of Substitutive Dental Sciences, College of Dentistry, Imam Abdulrahman Bin Faisal University, Dammam, Saudi Arabia

**Keywords:** denture base, elastic modulus, flexural strength, polymethyl methacrylate, silicon dioxide nanoparticles

## Abstract

**Objective** The aim of this study was to evaluate the effects of the addition of low-silicon dioxide nanoparticles (nano-SiO _2_ ) on the flexural strength and elastic modulus of polymethyl methacrylate (PMMA) denture base material.

**Materials and Methods** A total of 50 rectangular acrylic specimens (65 × 10 × 2.5 mm ^3^ ) were fabricated from heat-polymerized acrylic resin. In accordance with the amount of nano-SiO _2_ , specimens were divided into the following five groups ( *n* = 10 per group): a control group with no added SiO _2_ , and four test groups modified with 0.05, 0.25, 0.5, and 1.0 wt% nano-SiO _2_ of acrylic powder. Flexural strength and elastic modulus were measured by using a 3-point bending test with a universal testing machine. A scanning electron microscope was used for fracture surface analyses. Data analyses were conducted through analysis of variance and Tukey’s post hoc test (α = 0.05).

**Results** Compared with the control group, flexural strength and modulus of elasticity tended to significantly increase ( *p* ˂ 0.001) with the incorporation of nano-SiO _2_ . In between the reinforced groups, the flexural strength significantly decreased ( *p* ˂ 0.001) as the concentrations increased from 0.25 to 1.0%, with the 1.0% group showing the lowest value. Furthermore, the elastic modulus significantly increased ( *p* ˂ 0.001) at 0.05% followed by 1.0%, 0.25%, 0.5%, and least in control group.

**Conclusion** A low nano-SiO _2_ addition increased the flexural strength and elastic modulus of a PMMA denture base resin.

## Introduction

Polymethyl methacrylate (PMMA) denture base has been the most commonly used material for removable prostheses since 1930. The popularity of PMMA is based on its various advantages, which include being light weight, inexpensive, biocompatible, and having good esthetics. 1 However, it has poor mechanical properties, which are considered the main cause of denture fractures. 2 To overcome this drawback, many attempts to improve the properties of denture base resins have been attempted through chemical modifications or changes to the polymerization cycle to produce high-impact resins with greater strength. 3 4 Another way to enhance the physical properties of PMMA is to include various additives and reinforcement materials by incorporating wires, fibers, and metallic oxides. 5 6 In addition, nanoparticles have been added to PMMA to improve its mechanical properties, including compressive and flexural strength. 7 For example, adding silicon dioxide nanoparticle (nano-SiO _2_ ) to PMMA enhances thermal and physical properties due to their high surface activity, strong interfacial interaction with organic polymers, and large specific surface areas. 5 8 9 10

Many studies have investigated that the addition of nano-SiO _2_ to PMMA and have recommended using low-concentration additions. 9 11 12 13 According to Cevik and Yildirim-Bicer, the addition of nano-SiO _2_ in high concentrations (ratios: 1.0 and 5%) to PMMA decrease its mechanical properties. 11 Sodagar et al also incorporated nano-SiO _2_ at 1.0 and 0.5% into PMMA for removable appliances and found a decrease in flexural strength. Moreover, this reduction was directly related to the concentration of nano-SiO _2_ . 9 Gad et al and Abushowmi et al also added nano-SiO _2_ (0.25, 0.5, and 0.75%) to the PMMA repair resin and found that the highest increase in flexural strength is observed with 0.25% compared with the higher concentrations. 12 13 Therefore, lower concentrations of nano-SiO _2_ into PMMA to assess mechanical properties have been recommended for further investigations. A recent study by Alzayyat et al found that the addition of low concentrations of nano-SiO _2_ (0.05, 0.25, 0.5, and 1.0%) to PMMA denture base resin has positive effects on *Candida albicans* adhesion, hardness, and contact angle, while translucency and surface roughness are adversely affected at higher concentrations. 14 A successful denture base must have adequate mechanical properties for patient satisfaction. 3 Flexural stress during mastication can break the denture base. Moreover, a gradual irregular pattern of bone resorption can make the denture base become supported by uneven alveolar ridges. 15 Furthermore, low impacts and decreased flexural strength of the PMMA denture base material may cause a denture fracture. 16 Thus, high machinal properties, like flexural strength, of the denture base material is mandatory in preventing denture fractures under masticatory loading. The modulus of elasticity plays an important role in material rigidity, with an increase in the value, there is a decrease in elastic deformation and thus greater rigidity of the material. 17 To prevent permanent deformation of denture base materials caused by continuous stress or strain during mastication, these materials should have a high level of elastic modulus. 18

Although previous studies have been done on nano-SiO _2_ , the flexural properties of denture base materials modified with low levels of nano-SiO _2_ have not been investigated. Therefore, this study was done to evaluate the flexural strength and elastic modulus of PMMA denture bases following the addition of low concentrations of nano-SiO _2_ . The null hypothesis of the study was that the incorporation of low concentrations of nano-SiO _2_ would not improve the flexural strength of the PMMA-modified denture base resin.

## Materials and Methods

**Fig. 1:**
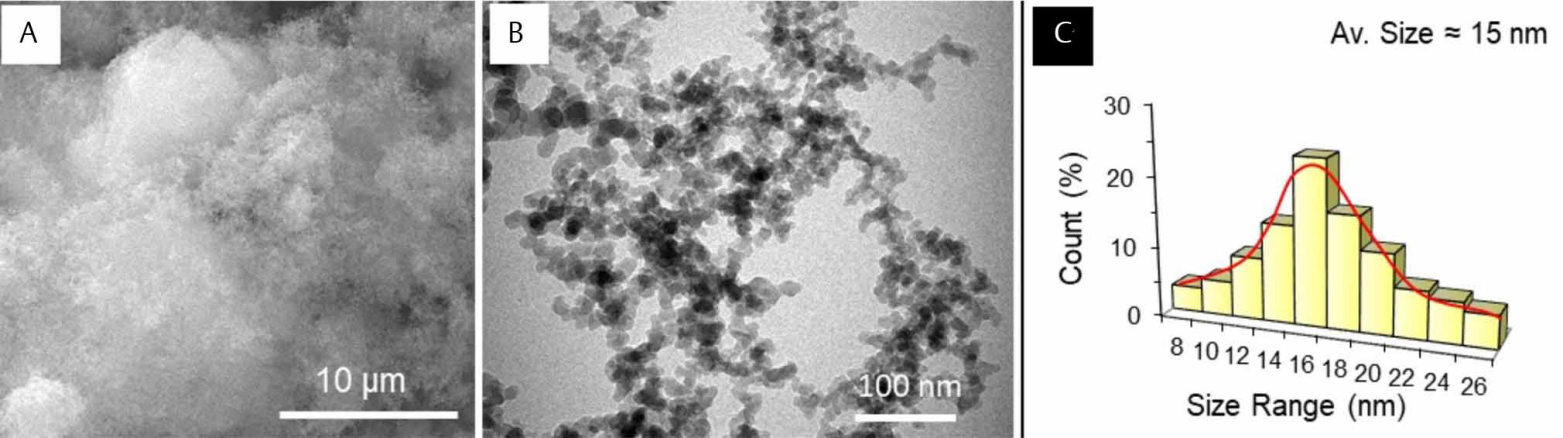
Representative ( **A** ) Scanning and ( **B** ) transmission electron microscopy and ( **C** ) size histogram of the nano-SiO _2_ particles. The nano-SiO _2_ particles were spherical in shape with an average size of approximately 15 nm. nano-SiO _2,_ silicon dioxide nanoparticles.

**Fig. 2:**
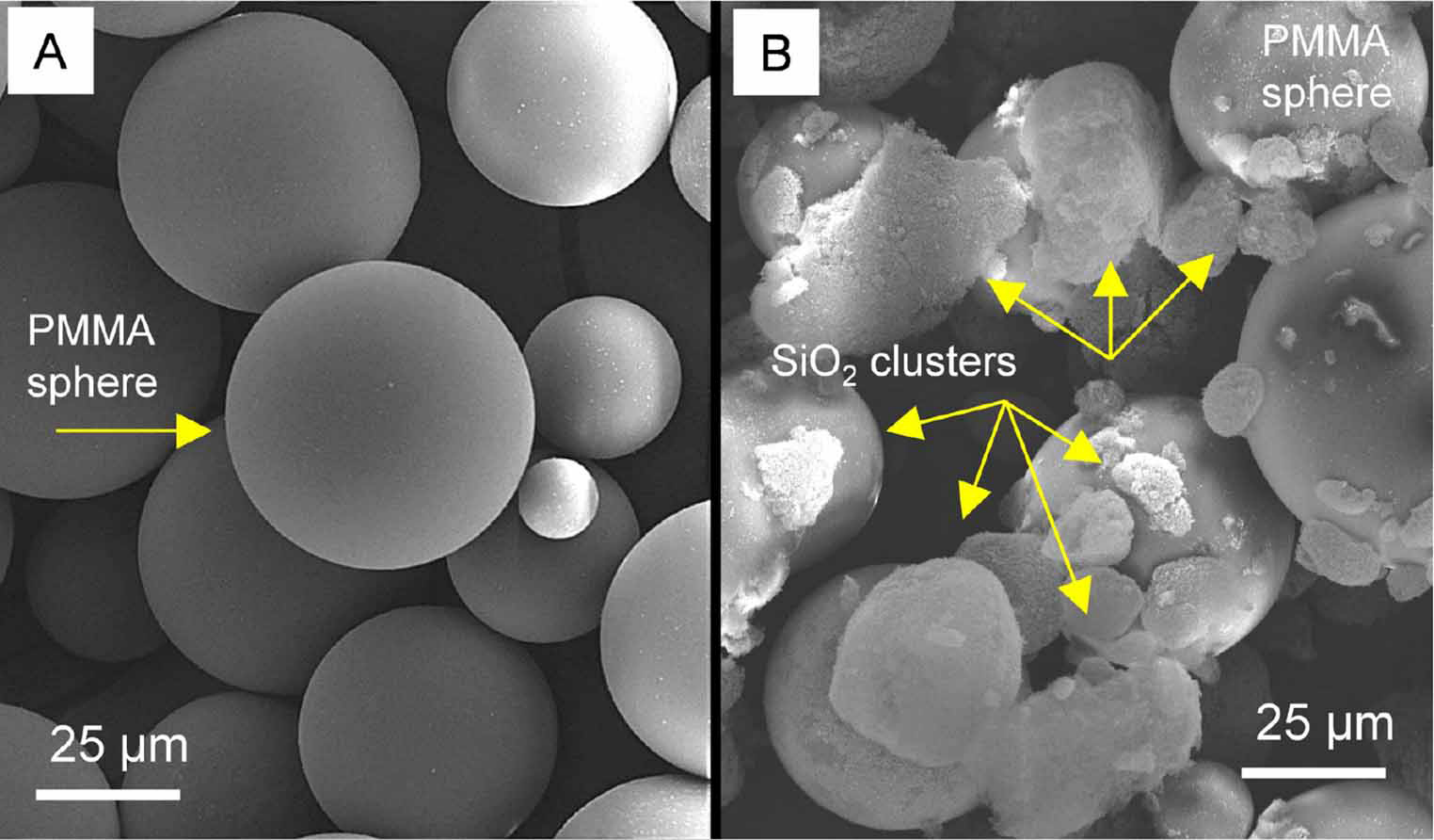
Scanning electron microscopy micrographs of the ( **A** ) pure PMMA and ( **B** ) a PMMA/nano-SiO _2_ mixture at the same magnification. A PMMA sphere and the clusters of nano-SiO _2_ particles are highlighted with arrows. The scale bars are 25 µm. PMMA, polymethylmethacrylate. nano-SiO _2,_ silicon dioxide nanoparticles.

The morphology and structure of the nano-SiO _2_ (AEROSIL R812; Evonik Degussa, Germany) were analyzed first by using a scanning electron microscope (SEM; Inspect S50; FEI, Brno, Czech Republic) and a transmission electron microscope (TEM; Morgagni 268; FEI, Brno, Czech Republic). The SEM and TEM were operated at an accelerated voltage of 20 kV (80 kV), respectively. The SEM and TEM analyses showed the characteristics and specifications of the nanoparticles ( Fig. 1 ). The nano-SiO _2_ particles exhibited spherical shapes with some degree of aggregation. Using the TEM, the average size of the particles was calculated at around 15 nm. Nano-SiO _2_ (white color; 99.5% purity; average size: 15 nm; specific surface area: 150–550 m ^2^ /g; and density: 2.2 g/cm) were then silanized with 3-(trimethoxysilyl) propyl methacrylate, (97%; γ-MPS) and silane (Shanghai Richem International, Shanghai, China) following the steps described by Karci et al and da Silva et al. 19 20 The silanized nano-SiO _2_ was weighed with a digital balance (WENSAR Mab Dab Series Analytical Balance, DAB 220) in concentrations of 0.05, 0.25, 0.5, and 1.0 wt% of the heat-polymerized acrylic powder 19 20 (Major Base 20; Major Prodotti Dentari SPA, Moncalieri, Italy). The concentrations of nano-SiO _2_ were independently blended with the PMMA powder at 400 rpm for 30 minutes to ensure equal filler distribution within the PMMA powder. Before heat polymerization, the mixture (PMMA/nano-SiO _2_ ) was evaluated by using the SEM to verify the uniform distribution of the nanoparticles within the PMMA powder ( Fig. 2 ). For comparison, the powder of the pure PMMA specimen was analyzed, which also measured the average diameter of the PMMA spheres at approximately 25 µm.

A total of 50 rectangular acrylic specimens with dimensions of 65 × 10 × 2.5 mm ^3^ were fabricated from heat-polymerized acrylic resin. In accordance with the amount of nano-SiO _2_ , specimens were divided into five groups ( *n* = 10 per group), a control group (with no additional nano-SiO _2_ ) and four study groups modified with 0.05, 0.25, 0.5, and 1.0 wt% nano-SiO _2_ of acrylic powder. Flexural strength and elastic modulus were measured by using a 3-point bending test with a universal testing machine. A SEM was used for fracture surface analysis. Data analyses were conducted through analysis of variance (ANOVA) and Tukey’s post hoc tests (α = 0.05). Acrylic resin specimens were prepared in a conventional method for denture base fabrication as described by Alzayyat et al. 14 Polishing was performed by using progressively finer, cylindrical silicon polishers (FINOPOL Polishers, 64830; LABOSHOP GmbH, Germany). To standardize the polishing procedure, it was performed by one investigator using a polishing cloth disk (TexMet C10 in, 42–3210, Buehler GmbH, Dusseldorf, Germany) and a mechanical polisher (Metaserve 250 grinder polisher; Buehler) at 100 rpm for 5 minutes in a wet environment. Specimens were stored in distilled water at 37°C for 48 hours prior to testing.

Flexural strength was measured by using a 3-point bending test on a universal testing machine (Electropuls E3000, Instron, United Kingdom). Each specimen was placed on the 3-point flexure apparatus where the support span was 50 mm. A load was applied at the midpoint of the prepared area with a crosshead speed of 5 mm/min until the specimen fractured, which is when the fracture load was recorded. The following formula was used to calculate the flexure strength values 21 :

FS = 3WL/2bh ^2^

Where FS is the flexural strength (MPa), W is the fracture load (N), L is the distance between the two supports, b is the specimen width, and h is the specimen thickness.

The elastic modulus was calculated by using the results from the flexural strength test using the following formula 22 :

E = FL ^3^ /4bh ^3^ d

Where E is the elastic modulus (MPa), F is the load (N) at a convenient point (p) in the straight line of the tension/deformation curve (elastic deformation), L is the distance between the two supports, b is the specimen width, h is the specimen thickness, and d is the deflection at point p.

**Fig. 3:**
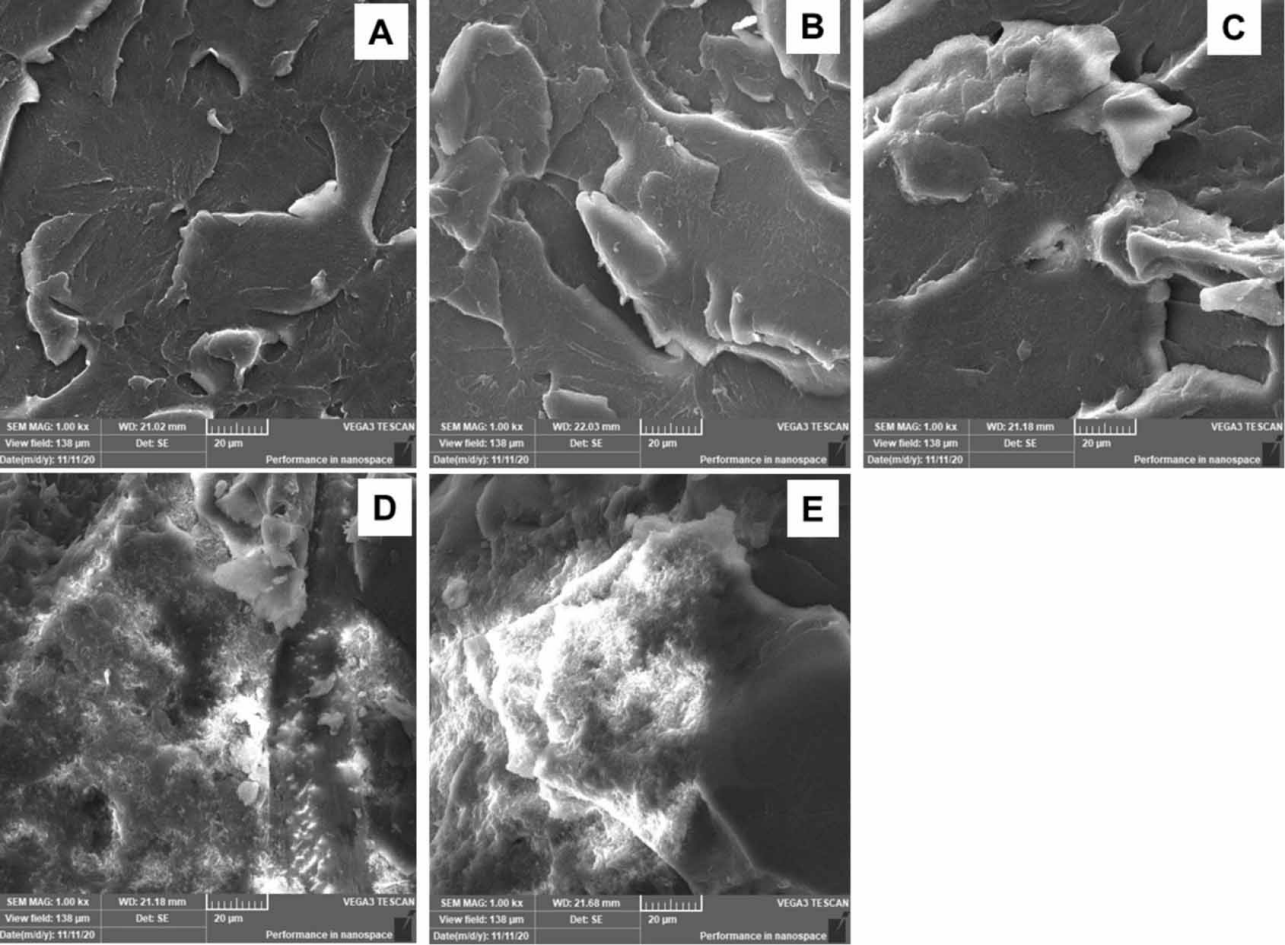
Representative scanning electron microscopy images of fractured specimens ( **A** ) unmodified, ( **B** ) 0.05% SiO _2_ , ( **C** ) 0.25% SiO _2_ , ( **D** ) 0.5% SiO _2_ , and ( **E** ) 1.0% SiO _2_ . nano-SiO _2,_ silicon dioxide nanoparticles.

After the flexural tests, the surface morphologies of the fractured specimens were analyzed by using the SEM. The SEM was operated at a medium accelerating voltage of 20.0 kV. For better image acquisition, the charging effects were minimized by coating the specimens with gold using a sputter coating machine (Quorum, Q150R ES, United Kingdom). The SEM micrographs of the control PMMA and PMMA/SiO _2_ /PMMA nanocomposite specimens were obtained at different magnifications to capture the important surface features and determine the mode of failure. The cross-sectional images of both the control and composite specimens are displayed at a representative magnification of ×1,000 ( Fig. 3 ). For a better comparison between different specimens, the electronic micrographs are shown at the same magnification.

The Statistical Package for Social Sciences (SPSS version 23)was used for data analysis. In the descriptive analyses, the means and standard deviations were calculated for each group. For inferential statistics, the normality of the data was tested first. Insignificant *p* -values from the Shapiro–Wilk test revealed that the data were normally distributed. Hence, a one-way ANOVA was used to test for any significance differences in mean values for flexural strength and elastic modulus at different concentration levels. In case of a significant ANOVA result, Tukey’s post hoc test was used for a pairwise comparison. The *p* -values less than 0.05 were considered statistically significant.

## Results

The variations for different nano-SiO _2_ concentrations on flexure strength and modulus of elasticity are presented in Table 1 . ANOVA results revealed that the variations in the mean elastic modulus and flexural strength due to changes in nano-SiO _2_ concentrations were significant. After performing statistical significance tests for flexure strength and elastic modulus, pairwise comparisons between the groups were tested through Tukey’s post hoc tests. The means, standard deviations, and significance levels of flexure strength and elastic modulus for varying nano-SiO _2_ concentrations are summarized in Table 2 . Our results showed that all tested groups had significantly higher flexural strength compared with the control group ( *p* ˂ 0.001). As such, the lowest flexural strength was with the control group (80.7 ± 1.75 MPa). Between the nano-SiO _2_ groups, there was a significant ( *p* ˂ 0.001) decrease in flexural strength, which was concentration dependent. As the concentration of nano-SiO _2_ increased, the flexural strength decreased, and at 1.0% nano-SiO _2_ the lowest flexural strength value (87.6 ± 1.4 MPa) was observed. For the elastic modulus, significantly increased ( *p* ˂ 0.001) at 0.05% with highest value (9,238.98 ± 86.1 MPa) followed by 1.0, 0.25, 0.5%, and least in control group. Pairwise comparisons of the elastic modulus between the control group and various concentrations of nano-SiO _2_ showed a significant increase in elastic modulus compared with the control group ( *p* ˂ 0.001). Between the nano-SiO _2_ groups, the 0.05% group had the highest elastic modulus value (9,238.98 ± 86.1 MPa). Pairwise comparisons between the group means found significant differences between all groups, with the exception of a nonsignificant difference between the 0.25% nano-SiO _2_ and 0.5% nano-SiO _2_ ( *p* = 0.121) groups. The highest elastic modulus was recorded with 0.05% (9,238.98 ± 86.1 MPa) followed by 1.0% nano-SiO _2_ (8,768.1 ± 96.0 MPa), while the lowest values were recorded with 0.5% nano-SiO _2_ and 0.25% nano-SiO _2_ with values of 8,184.9 ± 69.2 MPa and 8,268.7 ± 66.9 MPa, respectively, without a significant difference between these.

**Table 1:** Analysis of variance analysis of tested properties

	Groups	Sum of squares	df	Mean square	F-value	*p* -Value
Flexural strength	Between groups	2194.489	4	548.622	220.622	0.000 ^a^
	Within groups	111.902	45	2.487		
	Total	2306.391	49			
Elastic modulus	Between groups	41623604.984	4	10405901.246	1783.008	0.000 ^a^
	Within groups	262626.781	45	5836.151		
	Total	41886231.77	49			
^a^ Statistically significant at 0.05 level of significance.						

**Table 2:** Mean values, standard deviations, and significance levels between tested groups in relation to silicon dioxide nanoparticles concentrations

Groups	Flexural strength (MPa) Mean (SD)	Elastic modulus (MPa) Mean (SD)
Control	80.7 (1.75)	6540.1 (57.3)
0.05%	98.9 (1.77)	9238.98 (86.1)
0.25%	96.7 (1.43)	8268.7 (66.9) ^a^
0.5%	93.7 (1.5)	8184.9 (69.2) ^a^
1.0%	87.6 (1.4)	8768.1 (96.0)
Abbreviation: SD, standard deviation. ^a^ Nonsignificance between groups ( *p* ˃ 0.05).		

The representative SEM micrographs of the fractured unmodified and modified PMMA specimens (control = 0.05, 0.25, 0.5, and 1 wt%) are displayed in Fig. 1 . We observed that the surface morphology of the reinforced groups was greatly altered after the inclusion of nano-SiO _2_ into the PMMA matrices. These alterations seemed to be related to the increasing filler concentrations compared with the control group. As shown in Fig. 1 , the control specimen displayed a uniform lamellar structure with an absence of voids ( Fig. 1A ). Alternatively, the modified specimens (0.05, 0.25, 0.5, and 1 wt% SiO _2_ ) had some voids and nano-SiO _2_ particles in the form of clusters or agglomerations. The number of cluster particles and the size of the clusters increased with the increase in nano-SiO _2_ concentration in the PMMA matrix. Large aggregations of nano-SiO _2_ particles were clearly seen with the two groups containing 0.5 and 1.0% of SiO _2_ ( Figs. 3D and E ). The lamellar structure that appeared in the 0.05 and 0.25% specimens looked similar to the control group. However, this changed slightly to a faint lamella for the 0.5% group and finished with a smooth background in the 1.0% SiO _2_ group. The appearance of a whitened contrast due to the nature of the nano-SiO _2_ (poor electrical conductivity) reflected a brittle fracture. The composite matrix layer around the nano-SiO _2_ particles became stress whitened for low concentrations of nano-SiO _2_ ( Figs. 3B and C ). This suggests that with the addition of low concentrations of nano-SiO _2_ , the PMMA matrix yielded to produce plastic deformation, which absorbed deformation energy to improve the flexural strength of the modified specimens. For the 0.5 and 1.0 wt% SiO _2_ , the modified PMMA fractured surface ( Figs. 3D and E ) showed that with an increased filler ratio, there was a decreased adhesion between the PMMA matrix and the nano-SiO _2_ particles, which resulted in a drop in strength.

## Discussion

This study evaluated the effects of the addition of low concentrations of nano-SiO _2_ on the flexural strength and elastic modulus of a final PMMA/SiO _2_ nanocomposite. Based on the results, both the elastic modulus and flexural strength were affected by nano-SiO _2_ additions. Therefore, the null hypothesis was rejected.

The results showed significant increases in flexural strength in comparison to the unmodified group. This finding agreed with Gad et al, 12 who found that the addition of nano-SiO _2_ in low concentrations significantly improved the flexural strength of repaired acrylic resins. The increase in flexural strength may be attributed to the homogeneous distribution of low concentrations of SiO _2._ In addition, it has been suggested that its surface design fills the interpolymeric chain space of the resin base and restricts their movement, which can increase the flexural strength. 12 Moreover, the silane enables nano-SiO _2_ to form strong bonds with the polymer matrix creating crosslink bonds that may inhibit the crack progression and increase the flexure strength. 23 Alternatively, the results of Mussatto et al found that loaded nano-SiO _2_ into a resin denture base reduces the flexural strength. However, this was not concentration dependent nor affected by the method used. No major differences have been observed with surface silanization in mechanical behavior. 24

Among the nano-SiO _2_ groups, there was a concentration-dependent decrease in flexural strength with higher concentrations of nano-SiO _2_ . This reduction can be explained by the agglomeration of nano-SiO _2_ , which helps in the formation of loosely attached clusters acting as a stress-concentrating center that subsequently decreases the flexural strength. 1 23 Also, the presence of voids may be responsible for decreasing the flexural strength, thus leading to fractures of the resin base due to these voids. 11 12 Karci et al 19 have investigated PMMA incorporated with various concentrations of nano-SiO _2_ (1.0, 3, and 5%) and found that the flexural strength increases with 1.0% in comparison with the control group, but decreased as nano-SiO _2_ concentrations increased further. The greatest decrease was at 5% nano-SiO _2_ , which corresponds with our results where the greatest decrease was at 1.0% nano-SiO _2_ , which was the highest concentration used in the present study. 19 Moreover, findings by Cevik and Yildirim-Bicer agree with this study’s results. This group reported that adding nano-SiO _2_ in higher concentrations reduces flexural strength in the 1.0 and 5% groups. These decreases are thought to be due to voids and porosity changes, as shown with SEM findings in silica-incorporated specimens, which result in a reduced flexural strength. 11

The mechanical properties of the final PMMA/SiO _2_ nanocomposite depends on the shape, size, concentrations, and interactions with the polymer matrix. 25 It has been reported that nano-SiO _2_ have the lowest density, and this results in an increased particle amount per unit area compared with other metal oxide (Al _2_ O _3_ and TiO _2_ ) nanoparticles of the same concentration. This suggests that more agglomerations on the surface of the specimens lower the mechanical properties in nano-SiO _2_ reinforced groups. 19 For example, higher concentrations of nano-SiO _2_ (5%) indicate a higher volume and lower density of nano-SiO _2_ in the matrix with more agglomeration. Therefore, lower concentrations of nano-SiO _2_ are recommended. 19

Maxillary dentures frequently have midline fractures due to continuous flexion of the denture. Thus, the denture base should have satisfactory elastic modulus and flexural strength properties to avoid fractures and permanent deformations. 26 27 According to the results of the present study, the elastic modulus increased at 0.05% compared with the control group and decreased at 0.25 and 0.5%. However, the obtained elastic modulus values were still above the minimum recommended values (2,000 MPa) set by the American Dental Association specifications. 28 29

At 1.0% nano-SiO _2_ , the elastic modulus increased, and this increase may have been due to zeta sizer results, the micron distance between the nanoparticles, which can considerably reduce the polymer chain immobilization effect. 30 In addition, a relatively large agglomeration forms if the distribution of nanoparticles is not optimal, which leads to areas of material without reinforced nanoparticles without an immobilization effect. Therefore, there is a possibility of crack propagation between the reinforced fields through the unreinforced material. This agrees with Balos et al who used different concentrations of nano-SiO _2_ . This group found that with higher concentrations of nanoparticles and better particle distribution with 2% nanoparticles, there is a higher elastic modulus compared with 1.5% nanoparticles, which may be due to a less convenient zeta potential. 30

Clinically, the PMMA/SiO _2_ nanocomposite in a low concentration (0.05%) is promising for the denture base in terms of increasing flexural strength and the elastic modulus. However, the durability of this nanocomposite with the effects of various aging procedures (immersion in water, denture cleanser, brushing, and thermal stressing) requires more investigations. One limitation of this study was testing the mechanical properties of one type of acrylic resin in specimens that did not resemble a complete denture configuration or the environment of the oral cavity in addition to the low sample size. Accordingly, it is recommended to study nanocomposites containing low concentrations of nano-SiO _2_ and different denture base resin materials with artificial aging factors on complete denture bases in conditions simulating the oral cavity.

## Conclusion

Within the limitations of this study, we conclude that the incorporation of low concentrations of nano-SiO _2_ into a PMMA denture base resin increased the flexural strength, and this increase was concentration dependent. A low concentration of nano-SiO _2_ (0.05%) is the preferable concentration that may increase the flexural and elastic modulus properties of modified denture base resins.
